# (1*H*-Imidazole-κ*N* ^3^){*N*-[1-(2-oxidophenyl-κ*O*)ethyl­idene]-l-phenyl­alaninato-κ^2^ *N*,*O*}copper(II)

**DOI:** 10.1107/S160053680802758X

**Published:** 2008-09-06

**Authors:** Yong-Jun Han, Gan-Qing Zhao, Xiao-Jun Zhao, Wen-Li Song, Jie-Li Shi

**Affiliations:** a School of Chemistry and Chemical Engineering, Pingdingshan University, Pingdingshan 467000, People’s Republic of China

## Abstract

In the title compound, [Cu(C_17_H_15_NO_3_)(C_3_H_4_N_2_)], the Cu^II^ atom is four-coordinated by two O atoms and the N atom of the tridentate Schiff base ligand, and one N atom from the imidazole ligand in a distorted square-planar geometry. In the crystal structure, mol­ecules are linked into dimers by inter­molecular N—H⋯O hydrogen bonds.

## Related literature

For related literature, see: Basu Baul *et al.* (2007 ▶); Casella & Guillotti (1983 ▶); Ganguly *et al.* (2008 ▶); Parekh *et al.* (2006 ▶); Plesch *et al.* (1997 ▶); Usman *et al.* (2003 ▶); Vigato & Tamburini (2004 ▶).
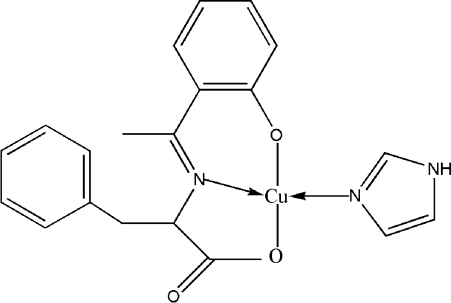


## Experimental

### 

#### Crystal data

[Cu(C_17_H_15_NO_3_)(C_3_H_4_N_2_)]
*M* *_r_* = 412.92
Orthorhombic, 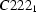

*a* = 16.8029 (16) Å
*b* = 19.8231 (19) Å
*c* = 11.3642 (11) Å
*V* = 3785.3 (6) Å^3^
*Z* = 8
Mo *K*α radiation
μ = 1.18 mm^−1^
*T* = 291 (2) K
0.43 × 0.34 × 0.25 mm

#### Data collection

Bruker SMART CCD area-detector diffractometer
Absorption correction: multi-scan (*SADABS*; Sheldrick, 1996 ▶) *T*_min_ = 0.630, *T*_max_ = 0.759
10101 measured reflections
3534 independent reflections
3008 reflections with *I* > 2σ(*I*)
*R*_int_ = 0.027

#### Refinement

*R*[*F* ^2^ > 2σ(*F* ^2^)] = 0.028
*wR*(*F* ^2^) = 0.067
*S* = 1.01
3534 reflections
245 parameters
H-atom parameters constrained
Δρ_max_ = 0.21 e Å^−3^
Δρ_min_ = −0.16 e Å^−3^
Absolute structure: Flack (1983 ▶), 1557 Friedel pairs
Flack parameter: −0.029 (12)

### 

Data collection: *SMART* (Bruker, 2000 ▶); cell refinement: *SAINT* (Bruker, 2000 ▶); data reduction: *SAINT*; program(s) used to solve structure: *SHELXS97* (Sheldrick, 2008 ▶); program(s) used to refine structure: *SHELXL97* (Sheldrick, 2008 ▶); molecular graphics: *SHELXTL* (Sheldrick, 2008 ▶); software used to prepare material for publication: *SHELXTL*.

## Supplementary Material

Crystal structure: contains datablocks global, I. DOI: 10.1107/S160053680802758X/ci2661sup1.cif

Structure factors: contains datablocks I. DOI: 10.1107/S160053680802758X/ci2661Isup2.hkl

Additional supplementary materials:  crystallographic information; 3D view; checkCIF report

## Figures and Tables

**Table:** 

Cu1—O1	1.8876 (19)
Cu1—N1	1.945 (2)
Cu1—O3	1.9511 (18)
Cu1—N2	1.958 (2)

**Table:** 

O1—Cu1—N1	93.33 (9)
O1—Cu1—O3	171.74 (9)
N1—Cu1—O3	84.90 (8)
O1—Cu1—N2	93.20 (9)
N1—Cu1—N2	162.84 (10)
O3—Cu1—N2	90.77 (8)

**Table 2:** Hydrogen-bond geometry (Å, °)

*D*—H⋯*A*	*D*—H	H⋯*A*	*D*⋯*A*	*D*—H⋯*A*
N3—H3*D*⋯O2^i^	0.86	1.95	2.789 (3)	166

Symmetry code: (i) 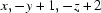.

**Fig. 1.**
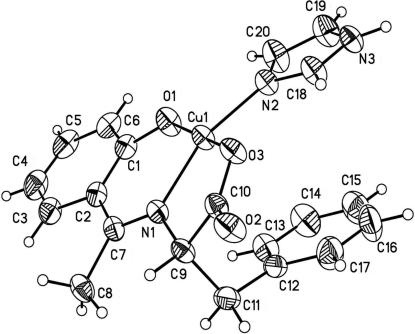
The molecular structure of the title compound, showing 30% probability displacement ellipsoids and the atom-numbering scheme.

**Fig. 2.**
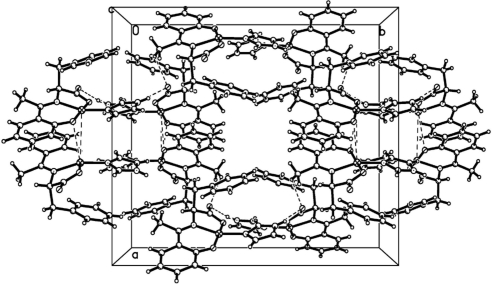
A view of the crystal packing along the c axis. Hydrogen bonds are shown as dashed lines.

## supplementary crystallographic information

### Comment

In the past decades, significant progress has been achieved in understanding the chemistry of transition metal complexes with Schiff base ligands composed of salicylaldehyde, 2-formylpyridine or their analogues, and α-amino acids (Vigato & Tamburini, 2004; Ganguly *et al.*, 2008; Casella & Guillotti, 1983). A few stuctural studies have been performed on Schiff base complexes derived from 2-hydroxyacetophenone and animo acids (Usman *et al.*, 2003; Basu Baul *et al.*, 2007; Parekh *et al.*, 2006). We report here the crystal structure of the title Cu^II^ complex.

The structure consists of discrete monomeric square-planar Cu^II^complex (Fig. 1 and Table 1). The four basal positions are occupied by three donor atoms from the tridentate Schiff base ligand, which furnishes an ONO donor set, with the fourth position occupied by one N atom from the imidazole ligand. The nitrogen heterocycle is planar and it forms an angle of 14.7 (2)° with the C1—C6 ring.

The crystal structure is stabilized by N—H···O type hydrogen bonds (Fig. 2 and Table 2). The H atom attached to N3 is hydrogen-bonded to the neighboring carboxylate oxygen O2 to form a dimer.

### Experimental

The title compound was synthesized as described in the literature (Plesch *et al.*, 1997). To *L*-phenylalanine (1.00 mmol) and potassium hydroxide (1.00 mmol) in 10 ml of methanol was added 2-hydroxyacetophenone (1.00 mmol in 10 ml of methanol) dropwise. The yellow solution was stirred for 2 h at 333 K. The resultant mixture was added dropwise to copper(II) acetate monohydrate (1.00 mmol) and imidazole (1.00 mmol) in an aqueous methanol solution (20 ml, 1:1 *v*/*v*), and heated with stirring for 2 h at 333 K. The dark blue solution was filtered and left for several days; the resulting dark blue crystals were filtered off, washed with water, and dried under vacuum.

### Refinement

All H atoms were positioned geometrically and refined as riding atoms, with C—H = 0.93 (CH) or 0.97 Å (CH_2_) and *U*_iso_(H) = 1.2*U*_eq_(C), C—H = 0.96 Å (CH_3_) and *U*_iso_(H) = 1.5*U*_eq_(C), and with N—H = 0.86 Å and *U*_iso_(H) = 1.2*U*_eq_(N).

### Crystal data

**Table:**

[Cu(C_17_H_15_NO_3_)(C_3_H_4_N_2_)]	*F*(000) = 1704
*M**_r_* = 412.92	*D*_x_ = 1.449 Mg m^−^^3^
Orthorhombic, *C*222_1_	Mo *K*α radiation, λ = 0.71073 Å
Hall symbol: C 2c 2	Cell parameters from 3925 reflections
*a* = 16.8029 (16) Å	θ = 2.4–23.2°
*b* = 19.8231 (19) Å	µ = 1.18 mm^−^^1^
*c* = 11.3642 (11) Å	*T* = 291 K
*V* = 3785.3 (6) Å^3^	Block, dark blue
*Z* = 8	0.43 × 0.34 × 0.25 mm

### Data collection

**Table:**

Bruker SMART CCD area-detector diffractometer	3534 independent reflections
Radiation source: fine-focus sealed tube	3008 reflections with *I* > 2σ(*I*)
graphite	*R*_int_ = 0.027
φ and ω scans	θ_max_ = 25.5°, θ_min_ = 2.4°
Absorption correction: multi-scan (SADABS; Sheldrick, 1996)	*h* = −20→16
*T*_min_ = 0.630, *T*_max_ = 0.759	*k* = −24→23
10101 measured reflections	*l* = −13→13

### Refinement

**Table:**

Refinement on *F*^2^	Secondary atom site location: difference Fourier map
Least-squares matrix: full	Hydrogen site location: inferred from neighbouring sites
*R*[*F*^2^ > 2σ(*F*^2^)] = 0.028	H-atom parameters constrained
*wR*(*F*^2^) = 0.067	*w* = 1/[σ^2^(*F*_o_^2^) + (0.0324*P*)^2^ + 1.1795*P*] where *P* = (*F*_o_^2^ + 2*F*_c_^2^)/3
*S* = 1.01	(Δ/σ)_max_ = 0.001
3534 reflections	Δρ_max_ = 0.21 e Å^−^^3^
245 parameters	Δρ_min_ = −0.16 e Å^−^^3^
0 restraints	Absolute structure: Flack (1983), 1557 Friedel pairs
Primary atom site location: structure-invariant direct methods	Flack parameter: −0.029 (12)

### Special details

**Table:**

Geometry. All e.s.d.'s (except the e.s.d. in the dihedral angle between two l.s. planes) are estimated using the full covariance matrix. The cell e.s.d.'s are taken into account individually in the estimation of e.s.d.'s in distances, angles and torsion angles; correlations between e.s.d.'s in cell parameters are only used when they are defined by crystal symmetry. An approximate (isotropic) treatment of cell e.s.d.'s is used for estimating e.s.d.'s involving l.s. planes.
Refinement. Refinement of *F*^2^ against ALL reflections. The weighted *R*-factor *wR* and goodness of fit *S* are based on *F*^2^, conventional *R*-factors *R* are based on *F*, with *F* set to zero for negative *F*^2^. The threshold expression of *F*^2^ > σ(*F*^2^) is used only for calculating *R*-factors(gt) *etc*. and is not relevant to the choice of reflections for refinement. *R*-factors based on *F*^2^ are statistically about twice as large as those based on *F*, and *R*- factors based on ALL data will be even larger.

### Fractional atomic coordinates and isotropic or equivalent isotropic displacement parameters (Å^2^)

**Table:**

	*x*	*y*	*z*	*U*_iso_*/*U*_eq_	
Cu1	0.10619 (2)	0.367615 (16)	0.77896 (3)	0.04364 (11)	
N1	0.13985 (13)	0.27457 (10)	0.80032 (19)	0.0406 (5)	
N2	0.10665 (16)	0.46531 (10)	0.75384 (17)	0.0459 (5)	
N3	0.13752 (15)	0.57138 (11)	0.7787 (3)	0.0547 (6)	
H3D	0.1544	0.6078	0.8115	0.066*	
O1	0.05861 (14)	0.34996 (10)	0.63167 (17)	0.0624 (6)	
O2	0.20326 (14)	0.32527 (10)	1.08596 (17)	0.0577 (6)	
O3	0.14173 (11)	0.37985 (8)	0.94100 (15)	0.0470 (5)	
C1	0.04519 (18)	0.29043 (15)	0.5868 (2)	0.0485 (7)	
C2	0.06894 (17)	0.22761 (15)	0.6360 (2)	0.0461 (7)	
C3	0.0429 (2)	0.16858 (17)	0.5793 (3)	0.0615 (9)	
H3	0.0564	0.1271	0.6117	0.074*	
C4	−0.0015 (2)	0.1697 (2)	0.4781 (4)	0.0778 (11)	
H4	−0.0192	0.1296	0.4447	0.093*	
C5	−0.0197 (2)	0.2304 (2)	0.4260 (3)	0.0704 (10)	
H5	−0.0475	0.2312	0.3553	0.084*	
C6	0.0028 (2)	0.28950 (16)	0.4780 (3)	0.0585 (8)	
H6	−0.0098	0.3301	0.4416	0.070*	
C7	0.11970 (16)	0.22199 (12)	0.7394 (2)	0.0444 (7)	
C8	0.15118 (19)	0.15280 (12)	0.7715 (3)	0.0580 (8)	
H8A	0.1901	0.1570	0.8328	0.087*	
H8B	0.1753	0.1325	0.7035	0.087*	
H8C	0.1081	0.1250	0.7984	0.087*	
C9	0.19625 (17)	0.27141 (13)	0.8989 (2)	0.0454 (7)	
H9	0.1897	0.2284	0.9404	0.054*	
C10	0.17829 (18)	0.32911 (13)	0.9830 (2)	0.0439 (7)	
C11	0.28234 (18)	0.27726 (15)	0.8539 (3)	0.0563 (8)	
H11A	0.3185	0.2738	0.9201	0.068*	
H11B	0.2933	0.2398	0.8014	0.068*	
C12	0.29811 (17)	0.34248 (14)	0.7897 (3)	0.0520 (7)	
C13	0.2807 (2)	0.34864 (17)	0.6704 (3)	0.0656 (10)	
H13	0.2606	0.3117	0.6296	0.079*	
C14	0.2928 (3)	0.4089 (2)	0.6120 (3)	0.0845 (12)	
H14	0.2813	0.4122	0.5321	0.101*	
C15	0.3216 (3)	0.4640 (2)	0.6713 (4)	0.0921 (13)	
H15	0.3292	0.5047	0.6321	0.110*	
C16	0.3390 (3)	0.45879 (18)	0.7879 (5)	0.0925 (14)	
H16	0.3589	0.4960	0.8281	0.111*	
C17	0.3274 (2)	0.39883 (19)	0.8466 (4)	0.0814 (11)	
H17	0.3396	0.3962	0.9263	0.098*	
C18	0.13541 (19)	0.51078 (14)	0.8262 (3)	0.0560 (9)	
H18	0.1524	0.5013	0.9024	0.067*	
C19	0.1082 (2)	0.56540 (16)	0.6691 (3)	0.0731 (10)	
H19	0.1025	0.5998	0.6140	0.088*	
C20	0.0885 (2)	0.49998 (17)	0.6544 (3)	0.0708 (11)	
H20	0.0660	0.4816	0.5868	0.085*	

### Atomic displacement parameters (Å^2^)

**Table:**

	*U*^11^	*U*^22^	*U*^33^	*U*^12^	*U*^13^	*U*^23^
Cu1	0.0632 (2)	0.03698 (15)	0.03072 (15)	0.00253 (17)	−0.00760 (17)	−0.00122 (14)
N1	0.0497 (13)	0.0383 (11)	0.0339 (13)	0.0008 (10)	0.0026 (10)	−0.0022 (9)
N2	0.0637 (15)	0.0421 (11)	0.0319 (12)	0.0013 (11)	−0.0068 (12)	0.0016 (9)
N3	0.0760 (18)	0.0383 (12)	0.0498 (14)	0.0005 (11)	−0.0040 (14)	0.0027 (12)
O1	0.0910 (16)	0.0539 (14)	0.0424 (11)	−0.0013 (11)	−0.0241 (11)	−0.0035 (10)
O2	0.0849 (16)	0.0513 (12)	0.0368 (10)	0.0122 (11)	−0.0180 (11)	0.0010 (9)
O3	0.0702 (13)	0.0376 (10)	0.0333 (10)	0.0094 (9)	−0.0094 (8)	−0.0010 (8)
C1	0.0508 (18)	0.0605 (19)	0.0342 (15)	−0.0049 (15)	0.0044 (13)	−0.0126 (14)
C2	0.0489 (18)	0.0522 (17)	0.0374 (15)	−0.0067 (14)	0.0086 (13)	−0.0135 (13)
C3	0.062 (2)	0.0598 (19)	0.062 (2)	−0.0079 (16)	0.0073 (17)	−0.0249 (17)
C4	0.070 (2)	0.084 (2)	0.080 (3)	−0.017 (2)	−0.001 (2)	−0.039 (2)
C5	0.057 (2)	0.109 (3)	0.046 (2)	−0.010 (2)	−0.0005 (17)	−0.030 (2)
C6	0.0519 (19)	0.084 (2)	0.0400 (19)	−0.0024 (19)	0.0000 (13)	−0.0078 (18)
C7	0.0538 (18)	0.0392 (13)	0.0400 (16)	−0.0032 (12)	0.0140 (13)	−0.0051 (11)
C8	0.078 (2)	0.0403 (15)	0.0562 (18)	0.0001 (13)	0.0123 (18)	−0.0059 (15)
C9	0.0586 (19)	0.0357 (14)	0.0419 (15)	0.0043 (13)	−0.0051 (14)	0.0034 (12)
C10	0.0550 (19)	0.0377 (15)	0.0391 (15)	−0.0003 (13)	−0.0068 (14)	0.0017 (12)
C11	0.056 (2)	0.0505 (18)	0.063 (2)	0.0117 (15)	−0.0050 (16)	−0.0008 (16)
C12	0.0463 (18)	0.0467 (16)	0.063 (2)	0.0048 (13)	0.0034 (17)	0.0049 (16)
C13	0.082 (3)	0.061 (2)	0.0542 (19)	−0.0013 (17)	0.0195 (18)	−0.0042 (16)
C14	0.115 (3)	0.079 (3)	0.059 (2)	−0.003 (2)	0.022 (2)	0.009 (2)
C15	0.111 (4)	0.065 (3)	0.100 (3)	−0.008 (2)	0.013 (3)	0.018 (2)
C16	0.116 (4)	0.055 (2)	0.107 (4)	−0.026 (2)	−0.029 (3)	0.010 (2)
C17	0.087 (3)	0.073 (2)	0.084 (3)	−0.011 (2)	−0.027 (2)	0.009 (2)
C18	0.088 (3)	0.0427 (16)	0.0375 (16)	0.0023 (15)	−0.0107 (15)	0.0019 (13)
C19	0.108 (3)	0.0540 (19)	0.057 (2)	−0.002 (2)	−0.013 (2)	0.0230 (16)
C20	0.115 (3)	0.0569 (19)	0.0401 (17)	−0.004 (2)	−0.025 (2)	0.0128 (15)

### Geometric parameters (Å, °)

**Table:**

Cu1—O1	1.8876 (19)	C7—C8	1.514 (4)
Cu1—N1	1.945 (2)	C8—H8A	0.96
Cu1—O3	1.9511 (18)	C8—H8B	0.96
Cu1—N2	1.958 (2)	C8—H8C	0.96
N1—C7	1.296 (3)	C9—C10	1.520 (4)
N1—C9	1.469 (3)	C9—C11	1.539 (4)
N2—C18	1.312 (4)	C9—H9	0.98
N2—C20	1.358 (4)	C11—C12	1.508 (4)
N3—C18	1.317 (4)	C11—H11A	0.97
N3—C19	1.346 (4)	C11—H11B	0.97
N3—H3D	0.86	C12—C17	1.381 (4)
O1—C1	1.305 (3)	C12—C13	1.392 (4)
O2—C10	1.246 (3)	C13—C14	1.383 (5)
O3—C10	1.271 (3)	C13—H13	0.93
C1—C2	1.422 (4)	C14—C15	1.371 (6)
C1—C6	1.427 (4)	C14—H14	0.93
C2—C3	1.405 (4)	C15—C16	1.361 (6)
C2—C7	1.457 (4)	C15—H15	0.93
C3—C4	1.371 (5)	C16—C17	1.377 (5)
C3—H3	0.93	C16—H16	0.93
C4—C5	1.376 (5)	C17—H17	0.93
C4—H4	0.93	C18—H18	0.93
C5—C6	1.365 (4)	C19—C20	1.349 (4)
C5—H5	0.93	C19—H19	0.93
C6—H6	0.93	C20—H20	0.93
			
O1—Cu1—N1	93.33 (9)	H8B—C8—H8C	109.5
O1—Cu1—O3	171.74 (9)	N1—C9—C10	108.6 (2)
N1—Cu1—O3	84.90 (8)	N1—C9—C11	110.4 (2)
O1—Cu1—N2	93.20 (9)	C10—C9—C11	109.8 (2)
N1—Cu1—N2	162.84 (10)	N1—C9—H9	109.3
O3—Cu1—N2	90.77 (8)	C10—C9—H9	109.3
C7—N1—C9	122.8 (2)	C11—C9—H9	109.3
C7—N1—Cu1	128.33 (19)	O2—C10—O3	124.3 (3)
C9—N1—Cu1	108.86 (16)	O2—C10—C9	118.5 (2)
C18—N2—C20	104.9 (2)	O3—C10—C9	117.1 (2)
C18—N2—Cu1	126.11 (19)	C12—C11—C9	113.0 (2)
C20—N2—Cu1	128.4 (2)	C12—C11—H11A	109.0
C18—N3—C19	106.8 (3)	C9—C11—H11A	109.0
C18—N3—H3D	126.6	C12—C11—H11B	109.0
C19—N3—H3D	126.6	C9—C11—H11B	109.0
C1—O1—Cu1	125.97 (19)	H11A—C11—H11B	107.8
C10—O3—Cu1	113.79 (16)	C17—C12—C13	117.4 (3)
O1—C1—C2	126.1 (3)	C17—C12—C11	122.0 (3)
O1—C1—C6	115.9 (3)	C13—C12—C11	120.6 (3)
C2—C1—C6	118.0 (3)	C14—C13—C12	120.8 (3)
C3—C2—C1	117.5 (3)	C14—C13—H13	119.6
C3—C2—C7	119.2 (3)	C12—C13—H13	119.6
C1—C2—C7	123.3 (2)	C15—C14—C13	120.2 (4)
C4—C3—C2	122.7 (4)	C15—C14—H14	119.9
C4—C3—H3	118.6	C13—C14—H14	119.9
C2—C3—H3	118.6	C16—C15—C14	119.6 (4)
C3—C4—C5	119.7 (3)	C16—C15—H15	120.2
C3—C4—H4	120.1	C14—C15—H15	120.2
C5—C4—H4	120.1	C15—C16—C17	120.4 (4)
C6—C5—C4	120.2 (3)	C15—C16—H16	119.8
C6—C5—H5	119.9	C17—C16—H16	119.8
C4—C5—H5	119.9	C16—C17—C12	121.5 (4)
C5—C6—C1	121.6 (3)	C16—C17—H17	119.3
C5—C6—H6	119.2	C12—C17—H17	119.3
C1—C6—H6	119.2	N2—C18—N3	112.3 (3)
N1—C7—C2	121.5 (2)	N2—C18—H18	123.9
N1—C7—C8	120.6 (3)	N3—C18—H18	123.9
C2—C7—C8	117.9 (2)	N3—C19—C20	106.8 (3)
C7—C8—H8A	109.5	N3—C19—H19	126.6
C7—C8—H8B	109.5	C20—C19—H19	126.6
H8A—C8—H8B	109.5	C19—C20—N2	109.2 (3)
C7—C8—H8C	109.5	C19—C20—H20	125.4
H8A—C8—H8C	109.5	N2—C20—H20	125.4

### Hydrogen-bond geometry (Å, °)

**Table:**

*D*—H···*A*	*D*—H	H···*A*	*D*···*A*	*D*—H···*A*
N3—H3D···O2^i^	0.86	1.95	2.789 (3)	166

Symmetry codes: (i) *x*, −*y*+1, −*z*+2.
